# Segregation Distortion Observed in the Progeny of Crosses Between *Oryza sativa* and *O*. *meridionalis* Caused by Abortion During Seed Development

**DOI:** 10.3390/plants8100398

**Published:** 2019-10-08

**Authors:** Daiki Toyomoto, Masato Uemura, Satoru Taura, Tadashi Sato, Robert Henry, Ryuji Ishikawa, Katsuyuki Ichitani

**Affiliations:** 1 United Graduate School of Agricultural Sciences, Kagoshima University, 1-21-24 Korimoto, Kagoshima, Kagoshima 890-0065, Japan; 2 Faculty of Agriculture, Kagoshima University, 1-21-24 Korimoto, Kagoshima, Kagoshima 890-0065, Japan; 3 Institute of Gene Research, Kagoshima University, 1-21-24 Korimoto, Kagoshima, Kagoshima 890-0065, Japan; 4 Graduate School of Agricultural Science, Tohoku University, Sendai, Miyagi 980-8577, Japan; 5 Queensland Alliance for Agriculture and Food Innovation, University of Queensland, Brisbane, Queensland 4072, Australia; 6 Faculty of Agriculture and Life Science, Hirosaki University, Hirosaki, Aomori 036-8561, Japan

**Keywords:** reproductive barrier, segregation distortion, abortion, wild rice, *O. meridionalis*, *O. sativa*, gene duplication

## Abstract

Wild rice relatives having the same AA genome as domesticated rice (*Oryza sativa*) comprise the primary gene pool for rice genetic improvement. Among them, *O. meridionalis* and *O. rufipogon* are found in the northern part of Australia. Three Australian wild rice strains, Jpn1 (*O. rufipogon*), Jpn2, and W1297 (*O. meridionalis*), and one cultivated rice cultivar Taichung 65 (T65) were used in this study. A recurrent backcrossing strategy was adopted to produce chromosomal segment substitution lines (CSSLs) carrying chromosomal segments from wild relatives and used for trait evaluation and genetic analysis. The segregation of the DNA marker RM136 locus on chromosome 6 was found to be highly distorted, and a recessive lethal gene causing abortion at the seed developmental stage was shown to be located between two DNA markers, KGC6_10.09 and KGC6_22.19 on chromosome 6 of W1297. We name this gene as *SEED DEVELOPMENT 1* (gene symbol: *SDV1*). *O*. *sativa* is thought to share the functional dominant allele *Sdv1-s* (s for *sativa*), and *O. meridionalis* is thought to share the recessive abortive allele *sdv1*-*m* (m for *meridionalis*). Though carrying the *sdv1*-*m* allele, the *O. meridionalis* accessions can self-fertilize and bear seeds. We speculate that the *SDV1* gene may have been duplicated before the divergence between *O. meridionalis* and the other AA genome *Oryza* species, and that *O. meridionalis* has lost the function of the *SDV1* gene and has kept the function of another putative gene named *SDV2*.

## 1. Introduction

Rice (*Oryza sativa*) is one of the most important staple crops in the world. It feeds about one-third of the world population. Wild rice relatives having the same AA genome as domesticated rice comprise the primary gene pool for rice genetic improvement and include the following species; *O. rufipogon*, *O. meridionalis*, *O. glumaepatula*, *O. barthii*, *O. longistaminata*. Another domesticated *Oryza* species *O. glaberrima* (African rice) also has an AA gemome, and contributes to rice improvement. Though there are several reproductive barriers among these species as described below, transfer of useful genes such as disease resistance gene from AA genome *Oryza* species to rice has been successful via hybridization.

*O. meridionalis* and *O. rufipogon* are found in the northern part of Australia [1]. *O. rufipogon* is inferred to be the direct progenitor of *O. sativa* [2], and widely distributed not only in Australia but also in South and South East Asia and New Guinea. On the other hand, the distribution of *O. meridionalis* is confined to the northern parts of Australia and Irian Jaya, Indonesia [1]. Molecular data provides support for the divergence of *O. meridionalis* from the other AA genome *Oryza* species [3,4,5,6,7]. This is reflected by low pollen fertility of the hybrids between *O. meridionalis* and the other AA genome species [8,9], with almost no progeny being produced from the selfing of the hybrids. To utilize the rice breeding potential of wild relatives of rice, a recurrent backcrossing strategy has been adopted to produce chromosomal segment substitution lines (CSSLs) carrying chromosomal segments from wild relatives of rice in the genetic background of cultivated rice [10,11,12,13]. Subsequent backcrossing with *O. sativa* as pollen parent was successful, because the F_1_ plants between *O. sativa* and its wild relatives retained female fertility.

To elucidate the genetic potential for the improvement of cultivated rice using these wild species, we produced three kinds of CSSLs with different Australian wild rice strains in the same genetic background. As a model agronomic trait, we selected late-heading, because the wild rice strains in this study head later than the recurrent parent Taichung 65 by about 50 days, and heading-time is easily scored. We have succeeded in mapping the late-heading time genes from these wild rice strains (see below) and found a new genetic distortion phenomenon in the *Oryza*. In this study, we report the genetic mechanism of the new distortion phenomenon.

## 2. Results

### 2.1. Mapping of Photoperiod Sensitivity Gene

**Figure 1:**
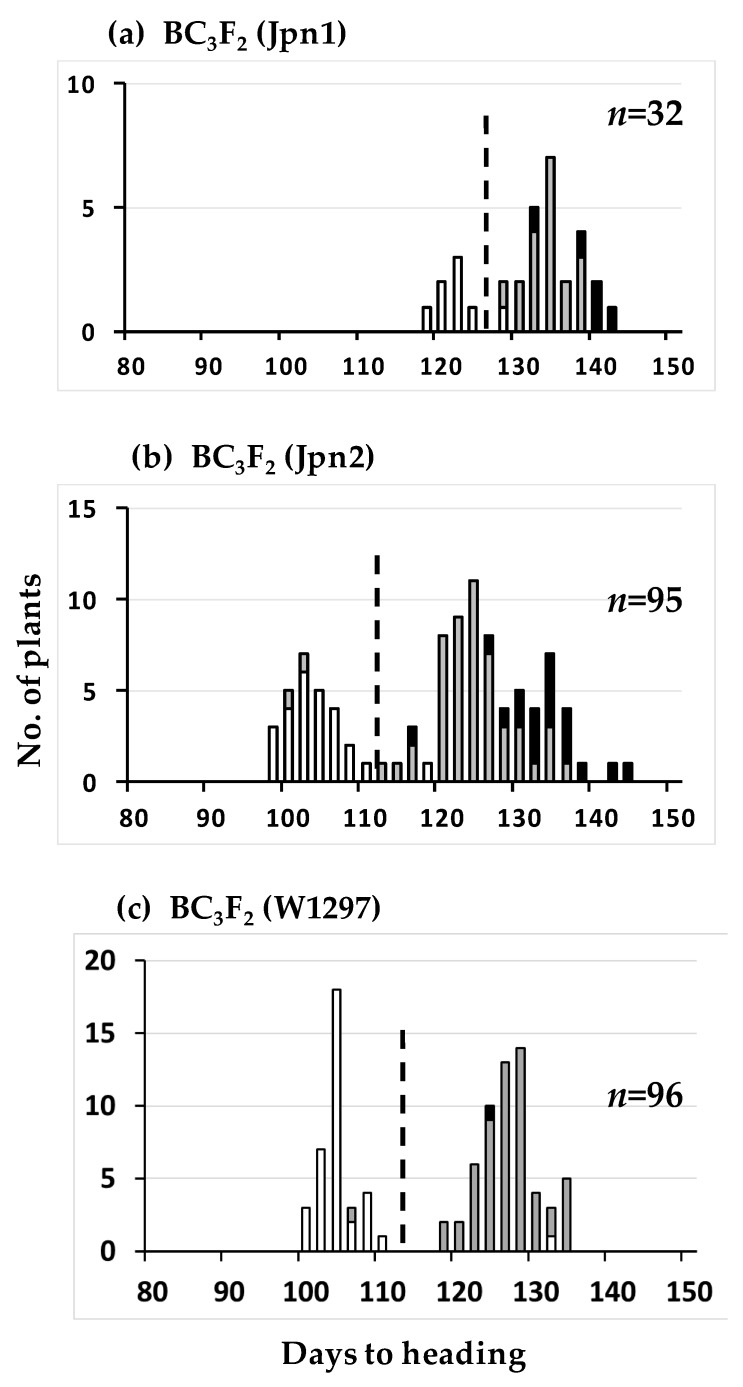
Frequency distributions of days to heading of the three BC_3_F_2_ populations using T65 as the recurrent parent. Jpn1, Jpn2, and W1297 were respectively used as donor parent in subfigure (**a**), (**b**), and (**c**). Three classified genotypes were assessed for RM136 as indicated: white, homozygous for T65, grey, heterozygous, black, homozygous for wild rice strains, Jpn1 (**a**), Jpn2 (**b**), and W1297 (**c**). Dotted lines dividing each population into early heading and late heading were drawn for chi-square analysis (see text).

Three wild rice strains, Jpn1, Jpn2, and W1297, and one cultivated rice cultivar Taichung 65 (T65) were used in this study. We bred various CSSLs in a T65 genetic background incorporating the three Australian wild rice strains, W1297, Jpn1, and Jpn2, chromosomal segments by recurrent backcrossing (see Material and Methods). Hereafter, the backcrossing populations using Jpn1, Jpn2, and W1297 as donor parent are described as BCnFm (Jpn1), BCnFm (Jpn2), and BCnFm (W1297), respectively. “n” and “m” represent numbers of backcrossing and selfing, respectively. The frequency distributions of days to heading of the three BC_3_F_2_ populations are shown in Figure 1. All populations showed bimodal distributions. A total of 94 DNA markers covering the whole 12 chromosomes and showing polymorphism between T65 and the three wild rice strains were subjected to preliminary linkage analysis using bulked DNA from the three BC_3_F_2_ populations. Only one marker RM136, located 568 kbp away from a photoperiod sensitivity gene *HD1* [14], showed heterozygosity in all the bulk DNAs. Chi square values for the independence between genotypes of RM136 and days to heading (early and late heading divided by the dotted line in Figure 1) were 26.880, 81.073, and 86.693 for Jpn1, Jpn2, and W1297, respectively, all highly significant (*P* < 0.0001). These results suggest that the three strains from Australia carry photoperiod sensitive alleles of the *HD1* locus, because heterozygotes and homozygotes of these strains at the RM136 locus headed much later than the homozygotes of T65. This cultivar proved to carry a photoperiod insensitive allele at the *HD1*(= *Se1*) locus [15], which behaved as an early heading-time allele in a usual cropping season in Japan [16,17,18].

In the BC_3_F_2_ (W1297), the segregation of the RM136 locus was highly distorted: very few homozygotes of W1297 appeared. To check if this phenomenon was specific to the cross with W1297 as donor parent, and to evaluate this phenomenon more clearly under a more uniform genetic background, BC_4_F_2_ populations with the same cross combinations were subject to further study. As for W1297, the BC_3_F_1_ plants producing the BC_3_F_2_ population for the analysis was backcrossed again to produce BC_4_F_1_ plants. Among them, late flowering plants were selected to produce BC_4_F_2_ populations. As for Jpn1 and Jpn2, different BC_3_F_1_ plants from that producing the BC_3_F_2_ population for the above experiment were backcrossed to produce BC_4_F_1_ plants. Among them, late flowering plants were selected to produce BC_4_F_2_ populations.

### 2.2. Mapping of Segregation Distortion Gene

**Figure 2:**
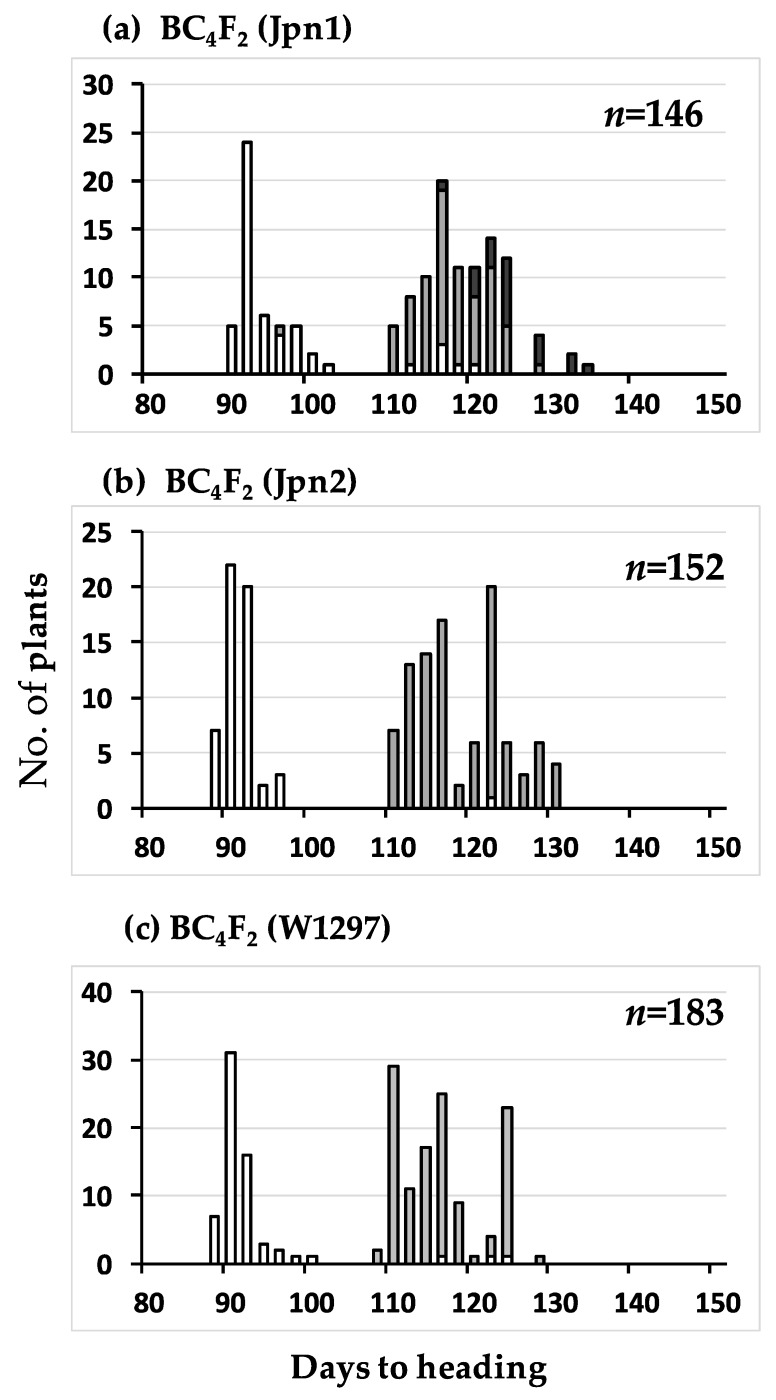
Frequency distributions of days to heading of the three BC_4_F_2_ populations using T65 as recurrent parent. Jpn1, Jpn2, and W1297 were respectively used as donor parent in subfigure (**a**), (**b**) and (**c**). Three classified genotypes were assessed for KGC6_12.02 as indicated: white, homozygous for T65, grey, heterozygous, black, homozygous for wild rice strains, Jpn1 (**a**), Jpn2 (**b**), and W1297 (**c**).

In the BC_4_F_2_ (W1297), the genotype of RM136 was distorted again (data not shown). In our preliminary experiment, among the published DNA markers around RM136, RM314 [19] located at 4,845kb, RM276 [19] at 6,231kb, RM7023 [20] at 6972kb, RM3628 [20] at 23,738kb and RM5314 [20] at 24,843kb on the IRGSP 1.0 pseudomolecule for chromosome 6 were fixed for the T65 allele. On the other hand, RM6818 and RM193 (Table 1) were segregating. These results suggest that the cause of segregation distortion is located between RM7023 and RM3628. Because other published DNA markers in our stocks failed in amplification of W1297 or did not distinguish T65 from W1297, we designed new DNA markers (Table 1), and performed linkage analysis. For the five consecutive markers from KGC6_12.02 to KGC6_19.48, only homozygotes of T65 and heterozygotes appeared (Figure 2), and no recombination occurred among the five markers (Table 2). The ratio of 64 homozygotes of T65: 119 heterozygotes fitted very well to 1:2 (*χ*^2^(1:2) = 0.221, *P* = 0.638).

**Figure 3:**
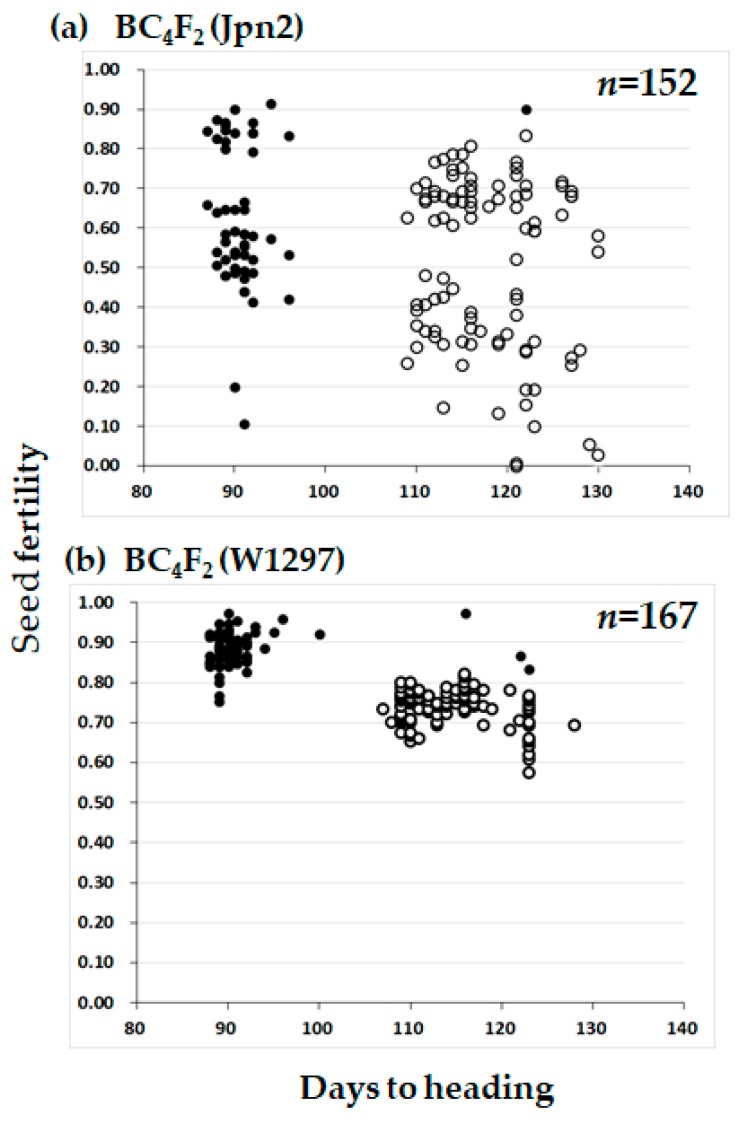
Scatter diagram of days to heading and seed fertility in the two BC_4_F_2_ populations using T65 as the recurrent parent. Jpn2 and W1297 were respectively used as donor parent in subfigure (**a**) and (**b**). Two classified genotypes were assessed for KGC6_12.02 as indicated: solid circle, homozygous for T65; open circle, heterozygous. In (**b**), plants used for testcross (Table 3) or damaged by birds in Figure 2 were removed in this figure.

**Figure 4:**
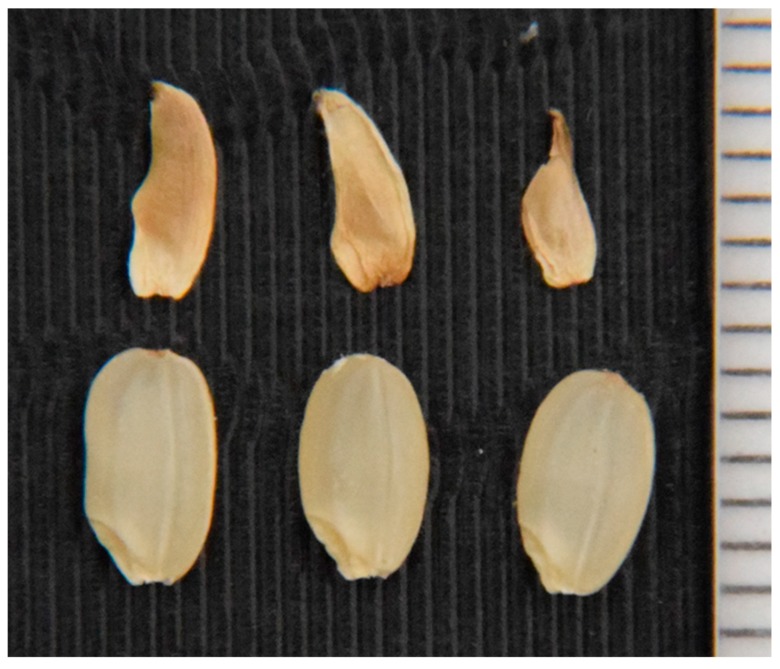
Sterile seeds aborted after fertilization (top) and normal fertile seeds (bottom) found in the BC_4_F_2_ (W1297). One unit of the rightmost scale indicates 1 mm.

**Figure 5:**
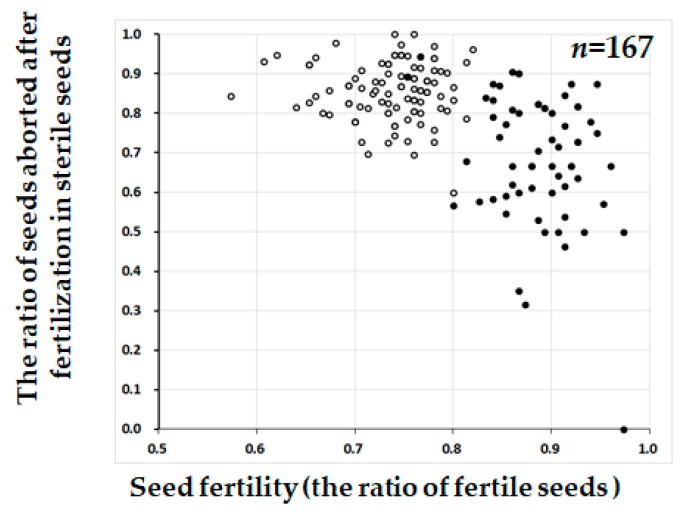
The scatter diagram between seed fertility (the ratio of fertile seeds) (X-axis) and the ratio of seeds aborted after fertilization in sterile seeds (Y-axis) in the BC_4_F_2_ population (W1297). Two classified genotypes were assessed for KGC6_12.02 as indicated: solid circle, homozygous for T65; open circle, heterozygous. Plants used for testcross (Table 3) or damaged by birds in Figure 2 were removed in this figure.

The distorted segregation ratio 1:2:0 can be explained by one pair of recessive lethal genes. If the lethality occurred at the seedling stage, about 25% of seedlings would be expected to die. However, our visual observation did not fit with such a phenomenon. We then speculated that segregation distortion occurred during seed development. If so, seed fertility of the heterozygotes should be lower than that of the T65 homozygotes by about 25%. To test this, we examined the seed fertility of each of the BC_4_F_2_ plants. In the BC_4_F_2_ (W1297) population, the heterozygotes for KGC6_12.02 showed lower seed fertility than the homozygotes of the T65 allele (Figure 3). If lower fertility was caused by one recessive gene, many of the sterile seeds were expected to be aborted after fertilization. Therefore, sterile seeds were dehusked to see if sterility occurred before or after fertilization (Figure 4). The proportion of seeds aborted after fertilization for heterozygotes for KGC6_12.02 was higher than that for homozygotes of the T65 allele (Figure 5). These results suggested that homozygotes of the W1297 allele for KGC6_12.02 die at the seed developmental stage.

### 2.3. Segregation Distortion Caused by Abortion During Seed Development

To confirm this hypothesis, the following experiments were performed. First, pollen fertility was examined for all BC_4_F_2_ (W1297) plants, with the result that all plants showed more than 90% pollen fertility (data not shown), suggesting that pollen sterility was not the cause of the distorted segregation ratio. Second, reciprocal backcrossing of heterozygotes for KGC6_12.02 to T65 to produce a BC_5_F_1_ generation was undertaken. The BC_5_F_1_ from both cross combinations showed the segregation ratio fitted a 1 heterozygote:1 homozygote ratio for the T65 allele (Table 3), indicating that normal gene segregation occurred at both the egg and pollen developmental stage. The BC_4_F_2_ plants used for backcrossing were also selfed to produce a BC_4_F_3_ generation. DNA was extracted from the embryo of the fertile seeds. The segregation ratio was largely distorted from 1:2:1 at the KGC6_12.02 locus, and no homozygotes for the W1297 allele for KGC6_12.02 appeared, indicating that segregation distortion occurred during seed development, and was not caused by ungerminated fertile seeds, though the segregation ratio did not fit to a 1:2:0 ratio. (Table 3). The BC_4_F_3_ plants deriving from selfed seeds of the BC_4_F_2_ plants heterozygous for the KGC6_12.02 locus also showed distorted segregation, and the ratio fitted a 1: 2: 0 ratio, confirming the other experimental results (Table 3). Taken together, all the experimental results indicated that a recessive lethal gene causing abortion at the seed developmental stage was located between KGC6_10.09 and KGC6_22.19 on chromosome 6 of W1297 (Table 2).

The same segregation distortion was also found in the BC_4_F_2_ (Jpn2) population (Figure 2). The ratio of 59 homozygotes of T65 allele: 97 heterozygotes at the KGC6_12.02 locus fitted very well to 1:2 (*χ*^2^(1: 2) = 0.556, *P* = 0.456), and no homozygotes of Jpn2 allele appeared. The seed fertility of heterozygotes of KGC6_12 was lower than that of the homozygote of T65 allele, supporting the view that a recessive lethal gene causing abortion at seed developmental stage was located close to KGC6_12 of Jpn2 (Figure 3). The seed fertility of BC_4_F_2_ (Jpn2) was highly variable for both homozygotes of the T65 allele and heterozygote at the KGC6_12.02 locus, suggesting that other genetic factor(s) were involved in the large variance of seed fertility. Our preliminary results showed low pollen fertility might be responsible for low seed fertility of some plants (unpublished data). Therefore, the cause of the seed sterility was not investigated further. For Jpn1, both BC_3_F_2_ (Jpn1) and BC_4_F_2_(Jpn1) showed that normal gene segregation occurred around the *HD1* locus (Figure 1 and Figure 2).

These results indicated that the two Australian *O. meridionalis* strains, W1297 and Jpn2, carry a recessive lethal gene causing abortion at the seed developmental stage, which was located between the two DNA markers, KGC6_10.09 and KGC6_22.19, spanning 12 Mb on chromosome 6, and that the Australian *O. rufipogon* strain Jpn1 does not carry such a gene.

## 3. Discussion

There have been many genes conferring hybrid seed sterility, hybrid pollen sterility, and segregation distortion found on *Oryza* chromosome 6 in inter-and intra-specific crosses, most of which *O. sativa* is involved with [21,22,23,24,25,26,27]. However, to our knowledge, the segregation distortion caused by seed abortion after fertilization has not been reported in the genus *Oryza*. We name this gene *SEED DEVELOPMENT 1* (gene symbol: *SDV1*), according to the gene nomenclature system for rice [28], because this gene is involved in the early seed developmental stage. In the intraspecific crosses among *O. sativa*, there have been no reports of gene distortion or partial seed sterility phenomena as described above on chromosome 6, though other phenomena have been reported [21,22,23,24,25,26,27]. Therefore, all *O*. *sativa* is thought to share the same functional dominant allele found in T65. This allele was called *Sdv1-s* (s for *sativa*). The homozygotes of the W1297 allele and the Jpn2 allele of this locus do not exist in the T65 genetic background probably because they die at the early seed development stage. W1297 and Jpn2 have originated from different places in Australia: W1297 is from Northern Territory, and Jpn2 is from Queensland. According to Juliano et al. [29], most crosses between Northern Territory and Queensland accessions produced sterile hybrids. Our preliminary results showed the hybrids from the reciprocal crosses between W1297 and Jpn2 were highly sterile (unpublished data). DNA marker-based analyses showed *O. meridionalis* genetic differentiation corresponding to geographic origin [29]. Further, in the CSSL lines of an *O. meridionalis* accession, W1625, chromosomal segments in a T65 genetic background [12], no lines were fixed for the W1625 chromosomal segment on which *SDV1* locus is located (https://shigen.nig.ac.jp/rice/Oryzabase/locale/change?lang=en). The results described above on the whole suggest that all *O. meridionalis* share the recessive abortive allele. This allele was called *sdv1-m* (m for *meridionalis*).

**Figure 6:**
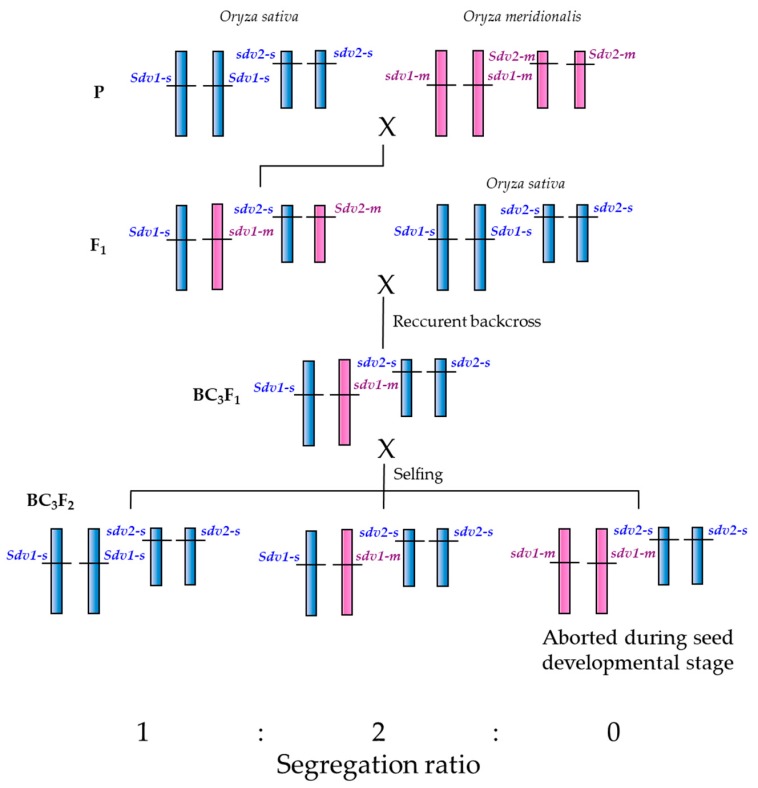
A genetic model that explains segregation distortion and seed sterility assuming gene duplication and loss of gene function for seed development.

Though carrying the recessive abortive allele in homozygous form at the *SDV1* locus, the *O. meridionalis* accessions can self-fertilize and bear seeds. We speculate that the *SDV1* gene may have been duplicated before the divergence between *O. meridionalis* and the other AA genome *Oryza* species, and that *O. meridionalis* has lost the function of the *SDV1* gene and has kept the function of the other gene while *O. sativa* kept the function of the *SDV1* gene and has lost the function of the other gene (Figure 6). Such duplication and loss of reproductive barrier-related genes has been reported; Yamagata et al. [30] found that the reciprocal loss of duplicated genes encoding mitochondrial ribosomal protein L27, essential for the later stage of pollen development, causes hybrid pollen sterility in F_1_ hybrid between *O. sativa* and *O. glumaepatula*. Nguyen et al. [31] reported that the duplication and loss of function of genes encoding RNA polymerase III subunit C4 hybrid causes pollen sterility in F_1_ hybrid between *O. sativa* and *O. nivara* (annual form of *O. rufipogon*). Ichitani et al. [32] performed linkage analysis of hybrid chlorosis genes in rice, and found that the causal recessive genes *hca1-1* and *hca2-1* are located on the distal region of the short arm of chromosome 12 and 11, respectively, known to be highly conserved as a duplicated chromosomal segment.

There are other models explaining the hybrid incompatibility (abortion, lethality, or weakness) known as the Bateson–Dobzhansky–Muller (BDM) model (for a review, Bomblies et al. [33]). In the incompatibility caused by the two nonallelic dominant genes, if the one locus is heterozygote or fixed for the incompatibility-causing allele, both heterozygotes and homozygotes of the incompatibility-causing allele of the other locus should show incompatibility. If the one locus is fixed for the normal allele, incompatibility does not occur. In the incompatibility caused by the heterozygote on one locus, only heterozygotes should show incompatibility. Therefore, these models cannot explain the segregation distortion in this study. In the hybrid breakdown model proposed by Oka [34], the combination of the heterozygotes or the homozygotes of recessive alleles at one locus and the homozygotes of recessive alleles at the other locus show incompatibility. This model cannot explain the segregation in this study either. Therefore, the gene duplication model as described above fits the phenomenon in this study best.

As the counterpart of *SDV1*, “the other” putative gene is named *SEED DEVELOPMENT 2* (gene symbol: *SDV2*). *SDV1* and *SDV2* are thought to be derived from duplication. *O. meridionalis* accessions and *O. sativa* accessions should carry the functional allele and the unfunctional allele at the *SDV2* locus, respectively. We name these respective alleles *Sdv2-m* and *sdv2-s*. The presence and chromosomal location of *SDV2* have not been elucidated. We are undertaking the genetic analysis of *SDV2*, tracing back to earlier backcrossing populations.

If useful genes of *O. meridionalis* for rice genetic improvement are located close to *sdv1-m*, the introgression of these genes into *O. sativa* genetic background should be combined with *Sdv2-m*. Therefore, the chromosomal location of *SDV2* and tightly linked DNA markers to it are urgently needed.

In the frequently cited high-density rice genetic linkage map by Harushima et al. [35], the centromeric region of chromosome six is located between 64.7 cM and 65.7 cM. Some of the DNA marker sequences located on the centromeric region are available in NCBI (https://www.ncbi.nlm.nih.gov). C574 (accession name: D15395) is located at 13,685 kb, and G294 (accession name: D14774) is located at 17,056 kb in Nipponbare genome (Os-Nipponbare-Reference-IRGSP-1.0). Therefore the physical size of the centromeric region is at least 3371 kb. The candidate chromosomal region of *SDV1* encompasses this region (Table 1 and Table 2). Recombination events were, in general, highly suppressed around the centromere. Our result is consistent with that. The combination of high resolution linkage analysis with gene expression analysis, gene disruption, and association study will be necessary to identify the *SDV1* gene.

Seed development is dissected into embryogenesis and endosperm development. We are undertaking microscopic observation of seed development of *Sdv1-s sdv1-m* heterozygotes in the T65 background to define the cause of the seed abortion. Several genes required for embryogenesis and endosperm development have been reported [36,37,38,39]. Identification of the *SDV1* and *SDV2* genes will contribute to the molecular genetics of seed development.

Direct evidence supporting the gene model was that the DNA from the embryo of aborted seeds deriving from the heterozygote of KGC6_1202 was homozygous for the W1297 allele. In our preliminary experiments, we tried to extract DNA from them, modifying the method below so that the DNA concentration would be higher. A few embryos were homozygous for the W1297 allele. However, PCR failed in most cases. This suggests that embryogenesis stops at an early stage in the homozygotes of *sdv1-m*. One alternative approach might be to extract DNA from developing seeds, not from mature seeds. Combination of microscopic observation of developing embryo and DNA genotyping will contribute to understanding the abortion mechanism caused by the *sdv1-m* gene. 

According to the chloroplast genome analyses by Wambugu et al. [40], Yin et al. [41] and Sotowa et al. [42], *O. rufipogon* in Australia carries a chloroplast genome similar to that of *O. meridionalis* rather than that of *O. rufipogon* in Asia and *O. sativa*, probably because of chloroplast capture (introgression). During the process of chloroplast capture, some nuclear genome genes could be shared by *O. rufipogon* in Australia and *O. meridionalis*. However, they might carry distinct alleles on *SDV1* and *SDV2* loci. When the DNA sequences of these alleles of the two loci are uncovered, this information can be applied for the analysis of plants growing in the wild, and possible ongoing hybridization between *O. rufipogon* and *O. meridionalis* can be monitored in the Northern part of Australia, in which the two species are sympatric. Hybrids have been found in the wild and confirmed by molecular analysis [43], but the low frequency of these hybrids and the continued existence of the two distinct AA genome taxa in the northern Australian environment may be explained by these genes that create a reproductive barrier.

## 4. Materials and Methods

### 4.1. Plant Material

Three wild rice strains, Jpn1, Jpn2, and W1297, and one cultivated rice cultivar Taichung 65 (T65) were used in this study. W1297 is a strain of *O. meridionalis* collected in Darwin, Northern Territory, Australia, and provided by National Institute of Genetics, Mishima, Japan. Jpn1 and Jpn2 were collected in Australia with the permission from the Queensland government, under the EcoAccess program [42]. Judging from its perennial life history, typical of Australian *O. rufipogon*, and Indel marker genotypes, Jpn1 was classified as *O. rufipogon*. The Australian *O*. *rufipogon* population at the Jpn1 site has been shown to have a chloroplast similar to that of *O*. *meridionalis* and a nuclear genome closer to *O*. *rufipogon* [44] suggesting it may need to be considered as a distinct taxon. Jpn2 was distinct with a short anther, typical of *O. meridionalis*, and perennial life history in its habitat in Queensland, Australia [42]. *O*. *meridionalis* is now described as including both annual and perennial types [45]. It had Indel marker genotypes that were the same as 18 *O. meridionalis* Core collection accessions. It was treated as a type of *O. meridionalis* based on five Indel DNA markers that reflect varietal differentiation in comparisons, such as Indica–Japonica, temperate Japonica–tropical Japonica with high accuracy [46,47]. Our visual observations indicated that the three wild rice strains each showed a uniform phenotype in the first growing year, suggesting that they had been fixed for at least the loci controlling agronomic traits. Before anthesis, the panicles of the wild rice strains were covered with bags made of glassine paper to force self-fertilization in every generation. The selfed progeny also showed uniform phenotypes. The preliminary analysis of DNA markers covering the whole 12 chromosomes indicated that they were homozygous at all the DNA marker loci. T65 is a Japonica cultivar used frequently in the study of rice genetics, as a recurrent parent of CSSLs, isogenic lines, and the study of induced mutation [12,21,48].

We bred CSSLs in a T65 genetic background incorporating the three Australian wild rice strains, W1297, Jpn1, and Jpn2, chromosomal segments by recurrent backcrossing. First T65 was crossed with W1297, Jpn1, and Jpn2 as pollen parents. One plant per each wild rice strain was used for producing the F_1_ generation. Then the F_1_ was backcrossed with T65 as a pollen parent in all subsequent backcross generations with some exception described above. A total of 39, 43, and 33 BC_1_F_1_ plants were obtained using W1297, Jpn1, and Jpn2 as donor parent, respectively. All the BC_1_F_1_ plants were backcrossed with T65. One BC_2_F_1_ plant originating from each BC_1_F_1_ plant was backcrossed with T65 to produce BC_3_F_1_. One BC_3_F_1_ plant originating from each BC_2_F_1_ plant was backcrossed with T65 to produce BC_4_F_1_. W1297, Jpn1, and Jpn2 have many characters different from T65, such as late heading, red pericarp, long awn, and easy shattering. Some BC_3_F_1_ plants had such characteristics in T65 genetic background, which was suitable for genetic dissection of these characters. As a model character, late heading was selected. We selected the latest BC_3_F_1_ plants and collected seeds from these plants to produce a BC_3_F_2_ generation. As shown above, because the segregation of genes conferring days to heading did not fit the expected Mendelian single gene segregation, we focused on the analysis of the distorted segregation. The BC_4_F_2_ generations deriving from the late heading BC_4_F_1_ plants were also examined. 

Plant cultivation followed Ichitani et al. [48]. Germinated seeds were sown in nursery beds in a greenhouse. About two weeks after sowing, seedlings were transferred out of the greenhouse. About 30 days after the sowing date, seedlings were planted in a paddy field at the Experimental Farm of Kagoshima University, Kagoshima, Japan. The fertilizers applied were 4, 6, and 5 g/m^2^, respectively, for N, K_2_O, and P_2_O_5_. Plant spacing was 15 × 30 cm. Sowing and transplanting were done respectively on May 31 and June 24 in 2015, on May 27and June 28 in 2016, May 25 and July 4 in 2017, respectively. Hybridization was performed as follows: For emasculation, panicles of the egg donor were soaked in hot water at 43 ℃ for 7 min. For pollination, the upper half of the open spikelets were cut about 30 min after emasculation. All the closed spikelets were cut off. Then pollen of the pollen donor was scattered on them. After pollination, panicles were covered with bags made of glassine paper. At least one panicle was left without pollination to check whether emasculation was complete.

### 4.2. Trait Evaluation

Heading date was recorded for each plant when the first developing panicle emerged from the leaf sheath of the flag leaf. Heading date was converted into days to heading. Seed fertility was evaluated by collecting 50 seeds from the upper side of each of the three panicles, using a modification of the method of Wan and Ikehashi [49], counting fertile and sterile spikelets on the upper half of 3–4 panicles for each plant. Seeds were scored as fertile or sterile. In the W1297 cross, sterile seeds were dehusked to see if sterility occurred before or after fertilization. The BC_4_F_2_ plants that produced the BC_5_F_1_ generation were dug up, and transferred from the paddy field to a glass house a day before pollination. We empirically know that rice plants undergoing such a treatment show lower seed fertility, probably because of root damage. Therefore, we did not evaluate seed fertility of these plants. Panicles of some plants were damaged by birds after heading. This is the reason for the inconsistency in BC_4_F_2_ plant number among tables and figures.

Pollen fertility of the BC_4_F_2_ (W1297) population was evaluated using iodine-potassium iodide solution. Panicles were collected about three days after emerging from the leaf sheath of the flag leaf, and dried in paper bags at room temperature. All the anthers in a spikelet collected one day before anthesis were cleaved to gather pollen on a glass slide. Pollen were stained with iodine-potassium iodide solution. More than 200 pollen grains were scored for each individual. Densely stained pollen with a normal size were scored as fertile. The other pollen were scored as sterile.

### 4.3. DNA Analysis

DNA from leaves and embryo from fertile seeds was extracted according to Ichitani et al. [48] with some modifications: Each leaf tip, 2.5 cm long from a single plant, or embryo from dehusked seeds was put in a well of a 96-deep-well plate. Then 100 μL of extraction buffer (100 mM Tris–HCl (pH 8.0), 1 M KCl, and 10 mM EDTA) was added with a 5-mm-diameter stainless steel ball to the well. After being covered with a hard lid, the plate was shaken hard (ShakeMaster ver. 1.2; BioMedical Science Inc., Tokyo, Japan) for 1 min to grind the leaves or embryos. After centrifuging, the plate was incubated at 70 °C for half an hour, then at room temperature for half an hour. Then 10 μL of the supernatant was recovered and 8 μL of 2-propanol was added. After centrifuging, the supernatant was discarded and the DNA pellet was rinsed with 50 μL of 70% ethanol. The DNA pellet was dried and dissolved in 50 μL of sterilized distilled water. It was very difficult to separate the embryo from the other part of seed completely. However, our preliminary experiment indicated that even if DNA was extracted from the whole dehusked seeds produced by a heterozygote for a DNA marker such as KGC6_12.02 (Table 1), DNA marker segregation was observed, suggesting that DNA from the parts of the dehusked seed other than the embryo was negligible. PCR mixture, cycle, electrophoresis, DNA staining, gel image documentation also followed Ichitani et al. [48].

### 4.4. DNA Markers

Most published PCR-based DNA markers for *Oryza* are based on an *O. sativa* genome sequence such as Nipponbare (Os-Nipponbare-Reference-IRGSP-1.0, [50]) and 9311 (GCA_0000046551, [51]). However, a preliminary survey comparing the genome of Nipponbare (IRGSP 1.0) and that of *O. meridionalis* accession (GCA_000338895.2. [7]) showed that there were many discrepancies between them, leading to expected failure in amplification from the *O. meridionalis* genome when using *O. sativa* genome-based DNA markers. Our strategy of designing co-dominant DNA markers was that insertion/deletion (indel) polymorphisms ranging from 5 to 100 base pairs were searched for between the Nipponbare and the *O. meridionalis* genomes. Then, the indels found only between *O. meridionalis* and Nipponbare, not between *meridionalis* and two Indica cultivars, 93-11 and HR-12 (GCA_000725085), were selected. The event causing such indels were thought to have occurred in Japonica rice after Japonica-Indica differentiation. T65 is a typical Japonica cultivar. Our preliminary survey showed that T65 shared the banding patterns of Nipponbare in most of the DNA markers examined [52]. Therefore, the indels as described above were expected to show polymorphism between T65 and *O. meridionalis*. The selected indels were screened based on sequence similarity surrounding indels between Nipponbare and the *O. meridionalis* genomes. The primer design followed Busung et al. [53].

## Figures and Tables

**Table 1:** Primer sequences of DNA markers designed or redesigned for linkage analysis of *SDV1* gene.

Marker Name	Kind of DNA Marker	Primer Sequence		Location on IRGSP 1.0 pseudomolecule chromosome 6		
				From	To	Source
KGC6_8.73	Indel	F	GAAGAGGAACATATGTGGTGTAAGC	8731826	8731914	This study
		R	AAAATTTATACTCTTGGTGACGTGA			
RM136	SSR	F	GAGAGCTCAGCTGCTGCCTCTAGC	8752461	8752562	Temnykh et al. [19]
		R	GAGGAGCGCCACGGTGTACGCC			
KGC6_8.82	Indel	F	TCTCTACCACACTCATCATCTGC	8820385	8820484	This study
		R	CCCTCGAGTAATAAACGATCCAG			
KGC6_10.09	Indel	F	TAGTCCTACGAAAACCCCTACTAGA	10090008	10090165	This study
		R	TTCCACGCACTAATACTACTACCTC			
KGC6_12.02	Indel	F	TTGATTTTGGGAAACATCAGGTAGC	12020476	12020625	This study
		R	AGCATGGTAATTTCATCGGATTCAA			
KGC6_13.00	Indel	F	CATTCGCATGGTAGCCTTTTCTTAT	13008017	13008169	This study
		R	CATAGGTGCCACAAGAGAAATCTTC			
RM6818	SSR	F	CGGCGAAGACTTGGAACCT	16582450	16582596	McCouch et al. [20]
		R	CCGTCACAAGGCTCGTCC			redesigned in this study
RM193	SSR	F	CAATCAACCAAACCGCGCTC	18086456	18086578	Temnykh et al. [19]
		R	CGCGGGCTTCTTCTCCTTC			redesigned in this study
KGC6_19.48	Indel	F	GAAGATAGTTAAGGGGTGTAGTGTGA	19483821	19484065	This study
		R	GACCAAAAGTTAAACAACATATTCTTCTAACCTAG			
KGC6_22.19	Indel	F	ACAAAATATGCTTTCTTCGTGCGTA	22191370	22191498	This study
		R	GCACTCAACTGTATCGTCTTTGAAA			

**Table 2:** Haplotypes around the segregation distortion region on rice chromosome 6 of BC_4_F_2_ (W1297).

Haplotype	Genotype of DNA Marker ^1^								No. of Plants
	KGC6_8.73	KGC6_10.09	KGC6_12.02	KGC6_13.00	RM6818	RM193	KGC6_19.48	KGC6_22.19	
1	H	H	H	H	H	H	H	H	115
2	T	T	T	T	T	T	T	T	59
3	H	H	T	T	T	T	T	T	2
4	T	T	T	T	T	T	T	H	2
5	H	T	T	T	T	T	T	T	1
6	H	H	H	H	H	H	H	H	1
7	W	H	H	H	H	H	H	H	1
8	W	W	H	H	H	H	H	H	1
9	H	H	H	H	H	H	H	W	1
									183

^1^ T, H, and W respectively denote homozygotes for T65, heterozygotes, and homozygotes for W1297.

**Table 3:** Segregation of progeny of BC_4_F_2_ (W1297) heterozygous for KGC6_12.02 genotype.

BC_4_F_2_			Genotype of the KGC6_12.02 ^1^																	
Individual Number			BC_4_F_3_											BC_5_F_1_						
			Plant					Embryo of Fertile Seeds						T65 as Pollen Parent				T65 as Egg Donor		
					*P*						*P*					*P*				*P*
					*χ* ^2^	*χ* ^2^					*χ* ^2^	*χ* ^2^				*χ* ^2^				*χ* ^2^
		T	H	W	(1:2:1)	(1:2:0)		T	H	W	(1:2:1)	(1:2:0)		T	H	(1:1)		T	H	(1:1)
1		10	23	0	0.004	0.712		21	26	0	0.000	0.099		10	17	0.178		12	13	0.841
2		12	14	0	0.004	0.166		17	27	0	0.000	0.456		10	17	0.178		8	9	0.808
3		12	20	0	0.004	0.617		16	30	0	0.000	0.835		17	10	0.178		16	7	0.061
4		18	18	0	0.000	0.034		15	33	0	0.000	0.759		12	12	1.000		14	15	0.853
5		10	15	0	0.011	0.480		20	27	0	0.000	0.180		10	14	0.414		15	13	0.705
6		12	18	0	0.005	0.439		21	27	0	0.000	0.126		14	12	0.695		14	9	0.297
7		6	23	0	0.002	0.149		24	24	0	0.000	0.014		8	20	0.023		21	23	0.763
																				
Sum		80	131	0	0.000	0.158		134	194	0	0.000	0.004		81	102	0.121		100	89	0.424

^1^ T, H, and W respectively denote homozygotes for T65, heterozygotes, and homozygotes for W1297.
